# *Sclerotinia* rot of *Zephyranthes candida* caused by *Sclerotinia sclerotiorum* and *Sclerotinia minor*

**DOI:** 10.3389/fmicb.2024.1414141

**Published:** 2024-07-09

**Authors:** Fuqiang Yin, Zhen Song, Lu Liu, Qin Xu, Jiamin Jiang, Zhien Xiao, Tiantian Guo, Yuxin Liu, Shaotian Zhang, Yue Yuan, Wanli Ma, Ming Liu

**Affiliations:** ^1^College of Biological and Food Engineering, Chongqing Three Gorges University, Chongqing, China; ^2^The Chongqing Engineering Laboratory for Green Cultivation and Deep Processing of the Three Gorges Reservoir Area’s Medicinal Herbs, Chongqing, China; ^3^Chongqing Three Gorges Vocational College, College of Agriculture and Forestry Science and Technology, Chongqing, China

**Keywords:** *sclerotinia* rot, *Zephyranthes candida*, multigene phylogeny, *Sclerotinia sclerotiorum*, *Sclerotinia minor*

## Abstract

*Sclerotinia rot* is a serious disease that occurs on *Zephyranthes candida*. A thorough understanding of the pathogenic fungal species and understanding the biological characteristics are important for controlling *sclerotinia*. Fungal strains were isolated from the diseased leaves of *Z. candida* through tissue isolation. Koch’s hypothesis screened pathogenic strains by pathogenicity of healthy leaves, including re-isolation and identification. A multigene phylogenetic tree was constructed by extracting genomic DNA from pathogenic strains and measuring the nucleotide sequences at four sites, including the internal transcribed spacer (ITS), RNA polymerase II second largest subunit (RPB2), glyceraldehyde-3-phosphate dehydrogenase (G3PDH), and heat shock protein 60 (HSP60). Morphological characteristics of the fungal structures were evaluated through microscopic analysis. The growth of pathogens was observed and recorded under different pH, different temperatures, different carbon sources and different nitrogen sources to clarify their biological characteristics. Representative strains D7, D13, X4, and X15 infected *Z. candida* and caused *sclerotinia* rot. At the beginning of the culture, white flocculent fungal hyphae appeared on the potato dextrose agar (PDA) medium, and black spherical to irregular-shaped sclerotia appeared at the edge of the colony after 7 days. The diameter of the sclerotia was 2.4–8.6 mm and 0.4–0.9 mm, respectively. One sclerotium was able to germinate from 1 to 5 apothecia. Ascus were cylindrical or spindle-shaped, with a size of 110.0–120.0 × 9.2–11.6 μm. One ascus contained eight colorless, oval ascospores, with a size of 8.4–12.0 × 4.5–5.5 μm. Based on the phylogenetic tree constructed with the gene sequences for ITS, G3PDH, HSP60, and RPB2, D7 and D13 shared 99% homology with *sclerotinia sclerotiorum*, whereas X4 and X15 shared 99% homology with *sclerotinia minor*. *S. sclerotiorum* growth was more suitable when the culture temperature was 15°C–25°C, pH 5.0, carbon source was maltose and nitrogen source was yeast powder. *S. minor* growth was more suitable when the culture temperature was 15°C, pH 5.0, the carbon source was glucose, and the nitrogen source was yeast powder. The results identified the pathogens as *S. sclerotiorum* and *S. minor*. To the best of our knowledge, this is the first report of *S. sclerotiorum* and *S. minor* causing *sclerotinia* rot on *Z. candida*. We herein aimed to identify the causal agent of *sclerotinia* rot of *Z. candida* in China based on morphological characteristics, molecular identification, and pathogenicity tests. Performed the experiments on the biological characteristics, to understand the law of disease occurrence. We also evaluated methods for the effective control of this disease. Our findings provide support for further studies on the pathogenesis mechanism of *sclerotinia* rot.

## 1 Introduction

*Zephyranthes candida* Herb. belongs to *Zephyranthes* Herb. and is also known as *Zephyranthes grandiflora*. It is an evergreen herbaceous ornamental plant with ecological benefit and ornamental value (Gong, 2019). It is widely used in city and small town landscaping and is an excellent garden plant (Liu et al., 2009). *Z. candida* is native to South America and is suitable for growth in warm areas. It is usually planted as garden ornamental plants in the southwest regions of China, including Chongqing, Sichuan, and Yunnan (Zhou, 2014; Wu and Liu, 2017). Currently, diseases reported on *Z. candida* include *Z. candida* anthracnose, which is caused by *Colletotrichum* spp. (Yu and Lin, 1998). Symptoms at the initial stage involve the leaves turning from green to yellow with irregular reddish-brown spots. At later stages, the diseased spots are enlarged and the leaves become dry and die. The whole process only takes about a few weeks. *Z. candida* red spot disease is caused by *Vermicularia* spp. (Wu and Zou, 1998). The initial signs are tiny red spots that expand into dark red and irregular disease spots. Finally, the entire disease spot becomes red, leading to death. White mildew of *Z. candida* is caused by *Sclerotina* spp. (Zhou and Qing l., 2011). In this disease, the leaves become yellow at the beginning, and white mycelium forms in the late stages, causing the entire plant to rot.

In recent years, *sclerotinia* diseases have become one of the most important diseases endangering *Z. candida*. Without timely control, *sclerotinia* diseases can cause mass death of *Z. candida*. Even a mild disease can greatly reduce the ornamental value of *Z. candida*. *Sclerotinia* rot, also referred to as white mold, stem rot, or crown rot (A and K, 2019), is a devastating necrotizing soil-borne disease (Lu et al., 2022) in which the leaves, flowers, fruits, and stems are compromised (Islam et al., 2021). This disease occurs worldwide and causes significant loss of important crops, vegetables, fruits, and ornamental plants (Florea et al., 2023). In China, *sclerotinia* rot results in the loss of 157,500 tons of rapeseed annually, accounting for 81.7% of the total rapeseed loss (Shang et al., 2024). In the United States, $560 million soybean losses were caused by *sclerotinia* rot in a particularly bad year (O’sullivan et al., 2021).

**Figure 1:**
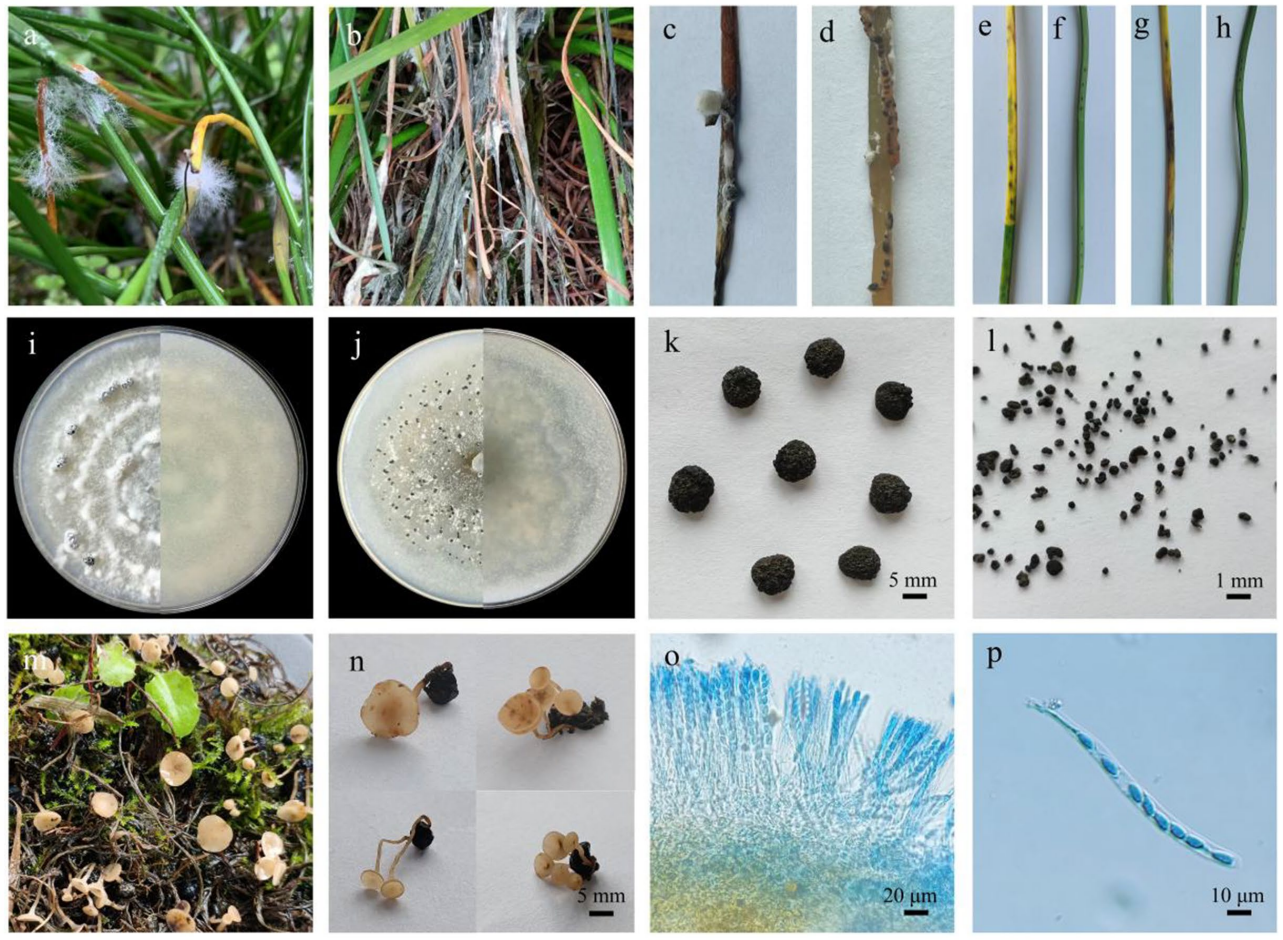
Symptoms of *Zephyranthes candida* infection caused by *Sclerotinia sclerotiorum* and *Sclerotinia minor*. **(A)** White aerial hyphae on *Zephyranthes candida*. **(B)** Disease symptoms in the field. **(C)** The large sclerotia of the dead *Zephyranthes candida* in the field. **(D)** The small sclerotia of the dead *Zephyranthes candida* in the field. **(E)** Symptoms of *Zephyranthes candida* leaves after the inoculation with strains D7 and D13. **(F)** Control group with strains D7 and D13. **(G)** Symptoms of *Zephyranthes candida* leaves after inoculating with strains X4 and X15. **(H)** Control group with strains X4 and X15. **(I)** *Sclerotinia sclerotiorum* (strains D7 and D13) morphology on PDA medium. **(J)** *Sclerotinia minor* (strains X4 and X15) morphology on PDA medium. **(K)** The sclerotia size of strains D7 and D13; bar = 5 mm. **(L)** The sclerotia size of strains X4 and X15; bar = 1 mm. **(M)** Apothecia formed under natural conditions. **(N)** One to five apothecia on a sclerotium; bar = 5 mm. **(O)** Cross section of apothecium and hymenium consisting of ascus and paraphyses; bar = 20 μm. **(P)** An ascus with eight ascospores; bar = 10 μm.

A disease with significant plant loss was detected in a *Z. candida* field in the Wanzhou area (30°32′N; 108°22′E) of Chongqing, China, in 2022 and subsequent years. In the beginning, the symptoms included a large number of white mycelia appearing on the leaves, which subsequently turned yellow (Figure 1A). Followed by plant withering and decaying in the field (Figure 1B), the white mycelium gathered into black sclerotia of two different sizes (Figures 1C,D). The symptoms were different from the disease symptoms reported in case of *Z. candida*, for example, *Colletotrichum* spp., *Vermicularia* spp., and so on. And the species of this pathogen could not be directly determined.

In this study, we combined morphological and multigene analyses based on internal transcribed spacer (ITS), RNA polymerase II second largest subunit (RPB2), glyceraldehyde-3-phosphate dehydrogenase (G3PDH), and heat shock protein 60 (HSP60) (Brauna-Morževska et al., 2023) to determine the pathogen species causing *sclerotinia* rot in *Z. candida*. The results of this study provide a reference for the prevention and control of *sclerotinia* rot in *Z. candida*.

## 2 Materials and methods

### 2.1 Fungal isolates and morphology

*sclerotinia*-infected plants were collected, and isolates were prepared from the tissue and sclerotia present on the surface of individual lesions. The diseased tissue segments (5-mm-long) and sclerotia were soaked in 75% alcohol for 30s and in 3% sodium hypochlorite solution for 3 min, rinsed three times with sterile distilled water, and dried on a sterile filter paper. They were plated onto potato dextrose agar (PDA) supplemented with 100 μg·mL^−1^ streptomycin sulfate (inhibit bacterial growth and prevent bacterial contamination of pathogens). The culture was purified using the hyphal-tip method (Gupta et al., 2015) and maintained on PDA dishes for culture, morphological, and pathological studies. The sclerotia were collected from the PDA medium and incubated for 2–5 weeks at temperatures ranging from 15°C to 25°C under high humidity conditions (over 85%) and all dark (Figure 1M). After carpogenic germination, the apothecia were sectioned and stained with aniline blue solution. The ascus and ascospores were observed under a microscope and photographed.

### 2.2 Pathogenicity test of the isolates

To confirm Koch’s postulates, the pathogenicity of the representative strains D7, D13, X4, and X15 was tested on healthy leaves of *Z. candida.* First, sterilized needles were used to make several small wounds on healthy *Z. candida* detached leaves on the ultra-clean workbench. Then, the mycelium was inoculated on the wound with a brush, sprayed with sterile water, and maintained at about 80% relative humidity. As a control, an equal amount of sterile distilled water was sprayed onto the *Z. candida* leaves with small wounds under the same humidity. After inoculation, the leaves were incubated under alternating dark (12 h) and light (12 h) conditions for 5 days. The pure cultures of the pathogen were re-isolated from the diseased *Z. candida* leaves and confirmed by a molecular analysis. The experiment was repeated three times.

### 2.3 DNA extraction and amplification

After incubating for 7 days, mycelia were collected and pathogen DNA was extracted using a fungal extraction kit. Primers for ITS1, ITS4, G3PDH, G3PDH, HSP60, and RPB2 were synthesized and used for Polymerasethe polymerase chain reaction (PCR). The PCR reactions contained 20 μL of 2x san Taq PCR Mix, 14 μL of ddH_2_O, 2 μL of genomic DNA, 1 μL forward primer, and 1 μL reverse primer. The total volume was adjusted to 40 μL. The specific gene regions and PCR primer sequences are listed in Table 1. The PCR conditions were as follows: initial denaturation at 94°C for 5 min, followed by 35 cycles of denaturation at 94°C for 30s, annealing for 30 s (54°C for ITS1 and ITS4 and 56°C for HSP60, G3PDH, and RPB2), extension at 72°C for 45 s, and a final extension for 10 min. The size of the PCR products was determined by 1% agarose gel electrophoresis along with standard DNA markers. The gels were stained with Gelgreen Nucleic Acid Gel Stain and photographed under UV light. The amplified products from four gene loci for the representative pathogens were purified and sent to Industrial Biotech (Chengdu) Co., Ltd. for DNA sequencing.

**Table 1:** Gene regions and PCR primers and programs used in this study.

Gene	Forward primers (for1)	Revers primers (rev1)
ITS	5´-TCCGTAGGTGAACCTGCGG-3′	5´-TCCTCCGCTTATTGATATGC-3′
HSP60	5´-CAACAATTGAGATTTGCCCACAAG-3′	5´-GATGGATCCAGTGGTACCGAGCAT-3
G3PDH	5´-ATTGACATCGTCGCTCTCAACGA-3′	5´-ACCCCACTCGTTCTCGTACCA-3′
RPB2	5´-CATTGGAAGTCTCGTCGTCA-3′	5´-ACCCCACTCGTTCTCGTACCA-3′

### 2.4 Phylogenetic analysis

The resulting sequences were blasted against the NCBI Database (https://blast.ncbi.nlm.nih.gov/Blast.cgi). ITS, G3PDH, HSP60, and RPB2 gene sequences were aligned with other *sclerotinia* sequences downloaded from the NCBI nucleotide database, including *Botrytis cinerea* as an outgroup taxon. Sequence alignment was performed via multiple alignment using fast fourier transform (Achilonu et al., 2023) and clustered using MEGA11 software employing the neighbor joining algorithm (Brauna-Morževska et al., 2023; Albert et al., 2024) to construct a phylogenetic tree.

### 2.5 Biological experiments

*S. sclerotiorum* and *S. minor* were treated with different temperatures, different pH, different carbon sources, and different nitrogen sources. The incubation temperatures were set to 10°C, 15°C, 20°C, 25°C, 28°C, 30°C, and 37°C, respectively. Use the standard titration solution of hydrochloric acid and sodium hydroxide to adjust the PDA medium with pH base of 5.0, 6.0, 7.0, 8.0, 9.0, and 10.0. The Czapek medium without carbon source was used and the carbon source in Czapek medium was replaced with starch, fructose, lactose, glucose, and maltose. The Czapek medium without nitrogen source was used as the control group, and the nitrogen source in the Czapek medium was replaced with urea, glycerol, ammonium chloride, protein, and yeast powder. *S. sclerotiorum* and *S. minor* mycelium blocks of 5 mm diameter were inoculated in the center of the medium with different treatments. And incubated in dark for 4 days with three replicates for each treatment, and colony diameter was recorded by Criss-cross method. The effects of different temperatures, different pH, different carbon sources and different nitrogen sources on the growth of pathogenic were observed and recorded.

## 3 Results

### 3.1 Morphological characterization of the isolates

The four isolated pathogenic strains had white primary hyphae, which grew thicker after 3–4 days and start to form white mycelium mass. After 7 days, the white mycelium mass completely changed into black sclerotia (Figures 1I,J). The diameter of the sclerotia of strains D7 and D13 was 2.4–8.6 mm (Figure 1K), whereas the diameter of the sclerotia of strains X4 and X15 was 0.4–0.9 mm (Figure 1L). One sclerotium was able to germinate from 1 to 5 apothecia (Figure 1N). The ascus were cylindrical or spindle-shaped, with a size of 110.0–120.0 × 9.2–11.6 μm (Figure 1O). One ascus contained eight colorless, oval ascospores with a size of 8.4–12.0 × 4.5–5.5 μm (Figure 1P). Previous studies have shown that *S. sclerotiorum* has the largest colonies and fastest growth rate, followed by *S. minor*, furthermore, the sclerotial size of *S. minor* is smaller than that of *S. sclerotiorum* (Deng et al., 2021). Based on growth rate, sclerotia size, and spore formation, D7 and D13 were believed to be *S. sclerotiorum*, and X4 and X15 were predicted to be *S. minor*.

### 3.2 Pathogenicity tests

After isolation and purification, four representative strains were collected, which included D7, D13, X4, and X15. The symptoms of the *Z. candida* leaves following inoculation with strains D7 and D13 are shown in Figure 1E. The symptoms of the *Z. candida* leaves inoculated with strains X4 and X15 are shown in Figure 1G. Both groups exhibited pathogenic symptoms that were identical to the diseased leaves observed in the field. In contrast, the leaves in the control group did not show any disease symptoms (Figures 1F,H). The pathogenicity test was repeated and confirmed three times. The strains were re-isolated and purified with the same pathogen as the inoculated diseased leaves to fulfill Koch’s postulate. Thus, we confirmed that the inoculated strain was the pathogen infecting the leaves of *Z. candida*.

### 3.3 Homology analysis of the pathogen sequences

**Figure 2:**
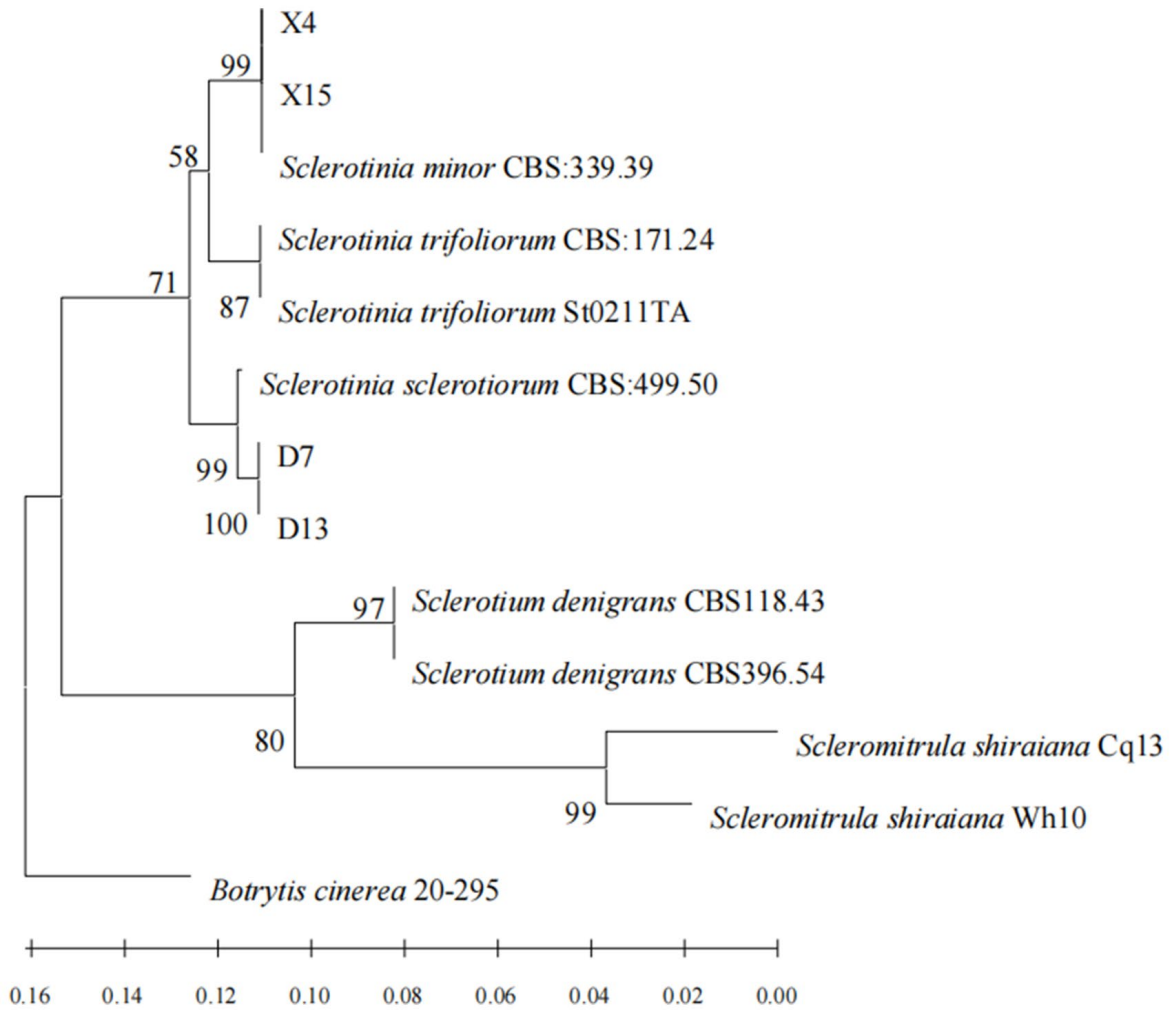
Phylogram of *sclerotinia* based on combined ITS, HSP60, G3PDH and RPB2 genes, with *Botrytis cinerea* (20–295) as outgroup (employing the‘Neighbor Joining’algorithm). The scale bar at the left–bottom represents 0.02 substitutions per site.

The PCR products of four representative isolates were sequenced and the nucleotide sequences were submitted for BLAST analysis using the NCBI-BLAST program. BLAST searches of the sequenced fragments revealed a match with the *S. sclerotiorum* and *S. minor* sequences. The sequences of the DNA regions amplified from strains D7, D13, X5, and X13 were deposited into GenBank and are included in the Table 2. In addition, a phylogenetic analysis of the four strains was conducted. The phylogenetic tree was constructed based on the ITS region, and the HSP60, G3PDH, and RPB2 gene sequences. Sequence alignment revealed that the sequences of X4 and X15 were 99% identical to *S. minor* CBS 339.39, whereas the sequences of D7 and D13 were 99% identical to *S. sclerotiorum* CBS 499.50 (Figure 2).

**Table 2:** Species and GenBank accession numbers of DNA sequences used in this study, with new sequences indicated in bold.

Species	Strain	Host	Country	GenBank accession number			
				ITS	HSP60	G3PDH	RPB2
*Sclerotium denigrans*	CBS118.43	*Convallaria majalis*	Germany	FJ231404	–	–	–
*Sclerotium denigrans*	CBS396.54	*Convallaria majalis*	Germany	FJ231405	–	–	–
*Sclerotinia trifoliorum*	CBS:171.24	–	United States	MF964318	KF878375	KF871408	–
*Sclerotinia trifoliorum*	St0211TA	*Trifolium ambiguum*	Poland	JQ743329	–	–	–
***Sclerotinia sclerotiorum***	**D7**	***Zephyranthes candida***	**China**	**OR511691**	**PP429289**	**PP429294**	**OR513110**
***Sclerotinia sclerotiorum***	**D13**	***Zephyranthes candida***	**China**	**OR758903**	**PP429290**	**PP429295**	**OR818445**
*Sclerotinia sclerotiorum*	CBS:499.50	–	Netherlands	MH856725	KF878370	KF871403	–
***Sclerotinia minor***	**X4**	***Zephyranthes candida***	**China**	**OR513111**	**PP429292**	**PP429296**	**OR511695**
***Sclerotinia minor***	**X15**	***Zephyranthes candida***	**China**	**OR758909**	**PP429293**	**PP429297**	**OR818446**
*Sclerotinia minor*	CBS:339.39	–	Canada	KF859929	KF878364	KF871397	–
*Scleromitrula shiraiana*	Cq13	Mulberry Fruits	China	MW013114	–	–	MW013884
*Scleromitrula shiraiana*	Wh10	Mulberry Fruits	China	MW013093	–	–	MW013863
*Botrytis cinerea*	20–295	Strawberry	United States	–	MZ288754	MZ288748	MZ288751

### 3.4 Biological characteristics

**Figure 3:**
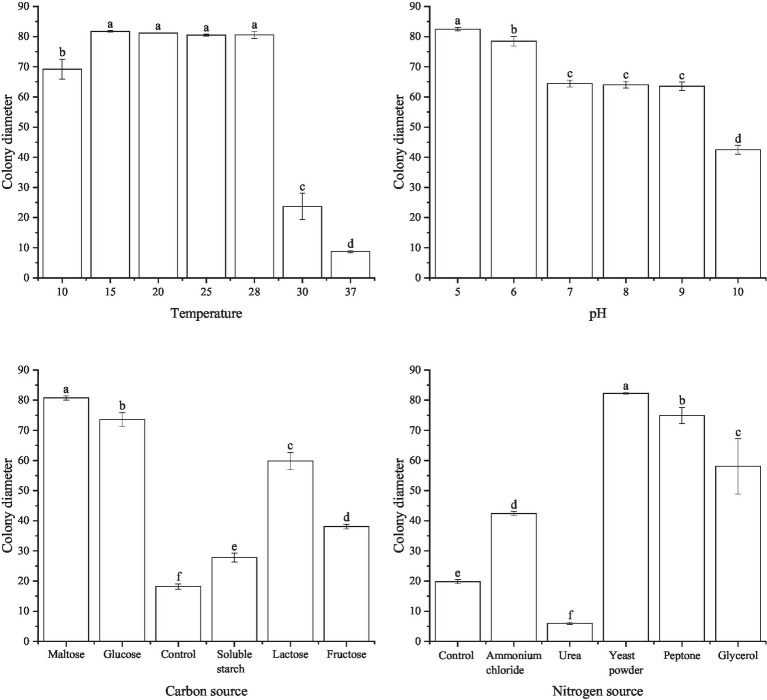
Effect of temperature, pH, carbon, and nitrogen sources on *Sclerotinia sclerotiorum.*

The experimental results indicated that the pathogen was capable of growing within a temperature range of 10°C–37°C. The highest mycelial growth rate of *S. sclerotiorum* was observed at 15°C, average colony diameter was up to 82.4 mm. At temperatures above or equal to 37°C, the mycelium begins to stop growth. *S. sclerotiorum* can grow at pH 5.0–10.0, but the growth rate varied. At pH of 5.0, the strains grew fastest and grew up to 55.2 mm. Hyphal growth was gradually slower with increasing pH. Under different carbon source conditions, When glucose was used as the carbon source, *S. sclerotiorum* hyphae grew faster, with a colony diameter of 42.5 mm after 4 days of culture. Next were maltose and lactose, with colony diameters of 34.3 mm and 24.7 mm. When starch and fructose were used as carbon sources, the hyphae grew slowly and were close to the control group. Under different nitrogen source conditions, When yeast powder was used as the nitrogen source, *S. sclerotiorum* mycelium grew faster with a colony diameter of 78.2 mm. Next by protein and glycerol, colony diameter 66.2 mm and 36.1 mm. When ammonium chloride was used as the nitrogen source, the colony diameter was close to the control group. When the nitrogen source was urea, the mycelium was barely grown (Figure 3).

**Figure 4:**
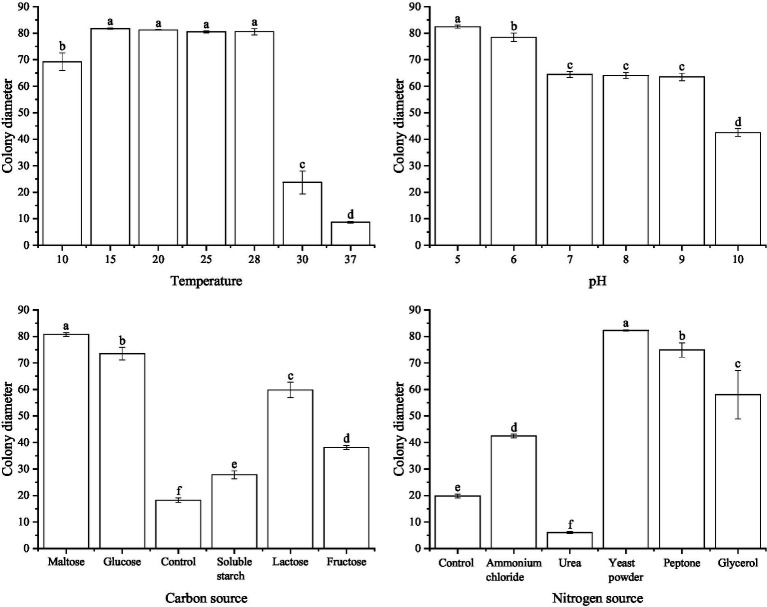
Effect of temperature, pH, carbon, and nitrogen sources on *Sclerotinia minor.*

At different temperature conditions, *S. minor* was able to grow normally at 10°C, with a colony diameter of 69.2 mm. At 15°C to 28°C, the colonies grew faster, at 81.8 mm, 81.2 mm, 81.0 mm and 80.5 mm, respectively. When the temperature reached 30°C, the hyphal growth began to be inhibited and the colony diameter was reduced to 23.7 mm. Temperature above or equal to 37°C, mycelium barely grew. Under different pH values, differences in the mycelial growth rate in *S. minor*. At pH 5.0, the strains grew the fastest, with a colony diameter of up to 82.5 mm. Hyphal growth was gradually slower with increasing pH. Under different carbon source conditions, when maltose was used as the carbon source, *S. minor* hyphae grew faster, with a colony diameter of 80.8 mm after 4 days of culture. Next by glucose and lactose, with colony diameter of 73.6 mm and 59.8 mm. Hyphe grew slowly with starch and fructose as carbon sources. Under different nitrogen source conditions, When yeast powder was used as the nitrogen source, *S. minor* mycelium grew faster with a colony diameter of 82.3 mm. Next by protein and glycerol, with colony diameters of 74.9 mm and 58.1 mm. With ammonium chloride as a nitrogen source. When the nitrogen source was urea, the colony diameter was lower than the control group, and the mycelium barely grew (Figure 4).

In summary, *S. sclerotiorum* has an optimum growth temperature of 15°C, an optimum pH value of 5.0, and the optimum carbon and nitrogen sources are glucose and yeast powder, respectively. *S. minor* has an optimum growth temperature was 15°C–28°C, the optimum pH is 5.0, and the optimum carbon and nitrogen sources were maltose and yeast powder, respectively.

## 4 Discussion

*sclerotinia* spp. is distributed globally and can cause white mold, stem rot, or crown rot on many crops and ornamental plants (Deng et al., 2021). The major species of phythopathological interest in the genus *sclerotinia* include *S. sclerotiorum*, *sclerotinia trifoliorum*, and *S. minor* (Marion and Myra, 2009). In the present study, the pathogens causing *sclerotinia* rot *Z. candida* were *S. sclerotiorum* and *S. minor*. *S. sclerotiorum* has a wide host range. It is a cosmopolitan plant pathogen that can infect more than 600 species of plants in 75 families, including leganaceae, solanaceae, and cruciferous. It is considered one of the most destructive plant pathogens (Li et al., 2023; Ruan et al., 2023). In nature, *S. sclerotiorum* can exist as mycelium, sclerotia, ascospores, or microconidia (Tang et al., 2011; Shang et al., 2024). *S. sclerotiorum* spends winter and summer in the form of sclerotia in the soil, plant residues, or seeds. The germination of sclerotia produces apothecia when climatic conditions are suitable. Apothecia are only produced within a very narrow temperature range (11–17°C), and even a few hours at 19 to 20°C will inhibit stipe formation (Crutcher et al., 2018). This results in the release of the ascospores, which spread with rain or airflow, and germinate as mycelial-infected plants (Lee et al., 2022). *S. minor* has a more restricted host range compared with *S. sclerotiorum*. It causes more than 90 types of plant infections (S et al., 1997; O’Malley, et al., 2015; Xu et al., 2023). *S. minor* primarily exists as mycelium and sclerotia. Plant infection occurs mainly through mycelium germination (S et al., 1997). The sclerotia size is one-tenth that of *S. sclerotiorum.* Carpogenic germination of *S. minor* and infection by ascospores is rare and has been seldom documented in most hosts (O’Malley, et al., 2015; Park et al., 2017; Mihajlović et al., 2022).

The occurrence and harm degree of the disease are greatly affected by the environment, such as temperature, pH, and nutrients (Zhang et al., 2024). The biological characteristics of pathogens are closely related to the regularity of disease occurrence. Most of *sclerotinia* have an optimal growth temperature of 20°C–25°C (Jiang et al., 2022; Zhou et al., 2023). This study shows that the optimal temperature range of *S. sclerotiorum* and *S. minor* ranges from 15°C to 28°C, which is more likely to occur in the early spring and winter climate in southern China. The pH in soil and air greatly affects the growth of *sclerotinia* (Xu et al., 2018). This study shows that the optimal pH value of *S. sclerotiorum* and *S. minor* is 5.0, more suitable for growth in an acidic environment. Carbon and nitrogen are essential nutrients for pathogenic fungi to survive in the host (Rabiul, et al., 2021). This study shows that pathogens have different degrees of assimilation of different carbon sources and different nitrogen sources. Among them, the pathogens have a strong utilization of glucose, maltose and yeast powder. However, urea has obvious inhibitory effect on pathogens and has potential control effect.

## 5 Conclusion

In this study, the pathogen of *Z. candida sclerotinia* disease was obtained by isolation and purification, and pathogenic strains were confirmed by pathogenicity tests. Morphology and multigene analysis based on ITS, HSP60, G3PDH and RPB2 identified pathogen species of *sclerotinia* disease were *S. sclerotiorum* and *S. minor*. This is the first documented occurrence to our knowledge of *sclerotinia* infecting *Z. candida* in China. *S. sclerotiorum* growth was more suitable when the culture temperature was 15°C–25°C, pH 5.0, carbon source is maltose and nitrogen source is yeast powder. *S. minor* growth was more suitable when the culture temperature was 15°C, pH 5.0, the carbon source was glucose, and the nitrogen source was yeast powder.

## Data availability statement

The datasets presented in this study can be found in online repositories. The names of the repository/repositories and accession number(s) can be found at: https://www.ncbi.nlm.nih.gov/genbank/, OR511691, PP429289, PP429294, OR513110, OR758903, PP429290, PP429295, OR818445, OR513111, PP429292, PP429296, OR511695, OR758909, PP429293, PP429297, OR818446.

## Author contributions

FY: Data curation, Methodology, Validation, Writing – original draft. ZS: Data curation, Methodology, Writing – original draft. LL: Investigation, Software, Writing – original draft. QX: Investigation, Software, Writing – original draft. JJ: Investigation, Software, Writing – original draft. ZX: Investigation, Validation, Writing – original draft. TG: Validation, Writing – original draft. YL: Investigation, Writing – original draft. SZ: Validation, Writing – original draft. YY: Validation, Writing – original draft. WM: Methodology, Validation, Writing – original draft. ML: Project administration, Resources, Writing – review & editing.
